# Cell-free plasma next-generation sequencing assists in the evaluation of secondary pneumonia in patients with COVID-19: a case series

**DOI:** 10.1017/S0950268823001711

**Published:** 2023-10-27

**Authors:** Joshua A. David, Bharadhwaj Kolipakkam, Megan K. Morales, Nicole C. Vissichelli

**Affiliations:** 1 Virginia Commonwealth University School of Medicine, Richmond, VA, USA; 2 Division of Hematology, Oncology and Palliative Care, Department of Internal Medicine, Virginia Commonwealth University, Richmond, VA, USA; 3 Division of Infectious Diseases, Department of Internal Medicine, Virginia Commonwealth University, Richmond, VA, USA

**Keywords:** cell-free microbial DNA, COVID-19, immunocompromised host, Karius, pneumonia

## Abstract

Secondary pneumonia occurs in 8–24% of patients with Coronavirus 2019 (COVID-19) infection and is associated with increased morbidity and mortality. Diagnosis of secondary pneumonia can be challenging. The purpose of this study was to evaluate the use of plasma microbial cell free DNA sequencing (mcfNGS) in the evaluation of secondary pneumonia after COVID-19. We performed a single-center case series of patients with COVID-19 who underwent mcfNGS to evaluate secondary pneumonia and reported the organisms identified, concordance with available tests, clinical utility, and outcomes. In 8/13 (61%) cases, mcfNGS detected 1–6 organisms, with clinically significant organisms identified in 4 cases, including *Pneumocystis jirovecii*, and *Legionella* spp. Management was changed in 85% (11/13) of patients based on results, including initiation of targeted therapy, de-escalation of empiric antimicrobials, and avoiding contingent escalation of antifungals. mcfNGS may be helpful to identify pathogens causing secondary pneumonia, including opportunistic pathogens in immunocompromised patients with COVID-19. However, providers need to carefully interpret this test within the clinical context.

## Background

Secondary bacterial and fungal infections complicate about 8–24% of cases of Coronavirus disease 2019 (COVID-19) and are associated with increased morbidity and mortality [1–5]. Like other respiratory viral infections, secondary bacterial infections are most common. However, there is also an increased risk of invasive mold infections (IMIs) after COVID-19, including COVID-19 associated pulmonary aspergillus (CAPA) and COVID-19 associated mucormycosis (CAM) [6]. While the mechanism behind these additional infections remains unclear, pathogen interactions with the immune system, damage to lung structures, empiric broad-spectrum antibiotic therapy, breaks in infection control measures, mechanical ventilation, and the use of immunomodulating agents likely contribute to the increased risk of bacterial infections while severe airway inflammation and relative immunosuppression contribute to the risk of CAPA [4, 7]. Patients with immunocompromising conditions at baseline are at even higher risk for superinfections, including opportunistic infections [8].

Early diagnosis and initiation of targeted therapies for secondary pneumonia are pivotal, however it is often difficult to distinguish from isolated severe or progressive acute respiratory syndrome coronavirus 2 (SARS-CoV-2) infection and to obtain a microbiologic diagnosis. Diagnosis is based on a combination of host factors, imaging, and microbiological testing including cultures, fungal biomarkers, polymerase chain reaction (PCR), and/or biopsy. Respiratory and tracheal aspirate cultures are limited by the feasibility of attaining quality sputum specimens and often have reduced sensitivity based on disease severity and empiric antimicrobial use [9, 10]. Bronchoalveolar lavage (BAL) and tissue biopsies permit direct detection of infectious organisms on culture or histopathology but are invasive, and results may be altered by empiric antimicrobial use [11, 12]. These procedures are also generally avoided in patients with active COVID-19 to limit the risk of healthcare worker exposure to aerosolized viral particulates [13]. Fungal biomarkers, including serum 1,3-β-D-Glucan (BDG) and galactomannan (GM), assist in the diagnosis of invasive aspergillosis. However, BDG is limited by a lack of specificity, and GM is limited by low sensitivity, which is lower in immunocompetent hosts and rapidly declines with empiric antifungal use [14].

Plasma microbial cell-free next-generation sequencing (mcfNGS) is a promising non-invasive approach for the diagnosis of infections, including pneumonia, with a fast turnaround time and the capability of detecting over 1,250 different organisms, including bacteria, DNA viruses, fungi, and eukaryotic parasites [15]. This test has previously shown utility for detecting opportunistic pathogens in immunocompromised hosts and it may have a unique role in the diagnosis of infections caused by pathogens that are difficult to identify and diagnose [16, 17]. Limited studies have investigated its clinical utility for the diagnosis of secondary pneumonia in the setting of COVID-19 [18, 19]. Herein we describe the use of mcfNGS to evaluate secondary pneumonia in patients with COVID-19.

## Methods

A retrospective single-center observational study was conducted from March 2020 to December 2021 at Virginia Commonwealth University Health System (VCUHS), an 811-bed tertiary care center, to evaluate patients with COVID-19 who were admitted to the hospital and underwent mcfNGS for the evaluation of superimposed lower respiratory tract infection. Patients over age 18 with severe acute respiratory syndrome coronavirus 2 (SARS-CoV-2) confirmed by polymerase chain reaction (PCR) during the study period were included. Patients were identified from a database of patients who had mcfNGS testing performed and had a positive SARS-CoV-2 PCR test. During the study period, universal screening for SARS-CoV-2 was performed at the authors’ institution. The VCUHS Pathology Laboratory utilized several different testing options during the study period, including a laboratory developed test which was performed on the Becton-Dickinson BD MAX system (Sparks, MD), as well as several commercially available tests used under the Food and Drug Administrations (FDA) Emergency Use Authorization (EUA). These EUA tests were manufactured by Cepheid (Sunnyvale, CA), Roche (Indianapolis, IN), BioFire (Salt Lake City, UT), and Thermo Fisher (Waltham, MA). COVID-19 infection was categorized by clinical severity according to the National Institutes of Health guidelines as asymptomatic (no symptoms present), mild (symptomatic without dyspnea or radiographic evidence of pneumonia), moderate (lower respiratory tract involvement with SpO2 ≥ 94%), severe (SpO2 < 94% or lung infiltrates >50%), or critical (acute respiratory distress syndrome, shock, and/or thrombotic disease associate with COVID-19) [20].

The diagnosis of secondary pneumonia was considered if patients had (1) clinical worsening due to fevers and/or new or worsening hypoxia in addition to (2) radiographic signs of new or worsening pulmonary infiltrates. If duplicate mcfNGS tests were performed, the first test was used. Peripheral blood samples were sent to Karius CLIA laboratory for mcfNGS (Karius, Inc., Redwood City, CA) at the discretion of the clinical provider. The indication for mcfNGS, patient history, antimicrobial use, mcfNGS results, correlation with serum biomarkers and cultures, clinical impact, and 30-day all-cause mortality were evaluated. Clinical impact was determined by independent review by two infectious diseases physicians of clinical documentation of decision making and changes to antimicrobial therapy after mcfNGS resulted. Pathogenicity was determined based on how the results correlated with available clinical, radiological, and laboratory data upon review of two independent Infectious Diseases providers. Data were collected and managed using REDCap electronic data capture tools hosted at Virginia Commonwealth University [21]. A descriptive analysis was performed using medians and ranges for continuous variables due to non-normal distributions and frequencies, and frequencies and percentages for categorical values. All analysis was performed using R (R Core Team, Vienna, Austria). This study was approved by the Virginia Commonwealth University Institutional Review Board prior to data collection. The data that support the findings of this study are available from the corresponding author, NV, upon reasonable request.

## Results

There were 13 patients with COVID-19 who developed clinical worsening with new or progressive radiographic findings and had mcfNGS performed to evaluate for secondary pneumonia after clinical worsening who were included in the study. Patient characteristics are summarized in Table 1. Twelve were immunosuppressed: 61% (8/13) following solid organ transplantation and 15% (2/13) with hematologic malignancies. The severity of COVID-19 infection was critical in 31% (4/13) patients, requiring mechanical ventilation in 2 and severe in 23% (3/13). There was a 25% (3/12) 30-day mortality in those with adequate follow up available. Forty-six percent (6/13) underwent bronchoscopy for further diagnostic workup. At the time of mcfNGS testing, 92% (12/13) were on antifungal therapy: 8 were on empiric treatment, 3 were escalated to combination therapy, and 3 were already on antifungals for other indications before the development of secondary pneumonia including prophylaxis (patients 3 and 7) and empiric treatment (patient 2). 9/13 (69%) patients were on antibacterial therapy, 8 were started on empiric treatment and 1 was already on antibiotics for other indications before the development of secondary pneumonia. The median (range) time on antimicrobial therapy before mcfNGS was 5 (1–682) days for antifungal agents and 5 (1–22 days) for antibacterial agents and the median time from secondary symptom onset to mcfNGS was 9 (4–37) days.

**Table 1.** Clinical variables of cohort patients who underwent mcfNGS testing^a^

Pt	Sex/Age	Underlying condition	Clinical finding	COVID severity (days)^b^	BAL result^c^	Fungal biomarkers	Days sx^d^	Antimicrobials (days)^e^	Infection (site, days)^f^	Organism (MPM) ^g^	Clinical impact	30-day mortality
1	F/28	OHT	Cavitary PNA	Severe (8)	–	BDG neg GM neg	8	AMB (5)	–	–	Stop AMB	Alive
2	F/53	OHT	Prolonged COVID with ongoing positivity, acute respiratory failure with mass-like infiltrates	Severe (110)	–	BDG neg GM neg	6	VCZ (35)	–	*Enterococcus faecalis* (1131), *Granulicatella adiacens* (405), *Rothia mucilaginosa* (3764)	Start treatment for *R. mucilaginousa;.* Prevent escalation of antifungals	Alive
3	F/64	Hx allo HSCT with recurrent AML	Acute hypoxic respiratory failure with nodular consolidation	Critical, HFNC (16)	–	BDG (349 pg./mL), GM neg	7	PSZ (39) MYC (1) PO VAN(22) Flagyl (22) LVX (8)	*Bacteroides ovatus* (B, 21), *Clostridioides difficile* (G, 19)	–	Stop MYC day mcfDNA returned	Died
4	M/28	OHT with active rejection	Worsening hypoxia with mixed consolidation and GGO	Mild (30)	GM neg Cx: *Candida spp* COVID (3)	BDG neg GM neg	4	IVCZ (3) FEP (2) PO VAN (7)	*C. difficile* (G, 7)	Torque teno virus (4268)	Stayed on IVCZ for ppx, stopped 10 days after mcfDNA resulted	Alive
5	M/60	CLL	Prolonged COVID-19, acute respiratory failure with worsening hypoxia	Critical (MV, shock) (27)	–ETT culture neg	BDG neg GM neg	15	MYC (5) VCZ (3) MEM (2)	*Escherichia coli* (U, 27)	–	No change in care due to severity of illness	Died
6	M/41	Acute alcoholic hepatitis	Tachypnea, consolidated GGO and reticulonodular opacities	Severe (11)	GM neg Cx: Rare yeast (11)	BDG (234 pg./mL), GM neg	10	MYC (8) FEP (8)	–	CMV (2054) EBV (360) *Lactobacillus rhamnosus* (254), *Saccharomyces cerevisiae* (196)	Listed for transplant. Remained on MYC for ppx given colonization	Alive
7	F/74	AML s/p allo HSCT c/b GVHD	New onset SOB with patchy GGO after prior COVID-19	Critical (136)	GM neg Cyto: rare fungal hyphae Cx: yeast DAH (11)	BDG neg GM 0.59 IU	11	PSZ (682) MYC (1) FEP (1)	–	*Enterococcus faecium* (1653)	Changed to VCZ/MYC given hyphae on cytology despite.	Alive
8	M/36	OHT	Acute respiratory failure with findings consistent with ALI and atypical infection	Critical, MV (29)	GM neg Cx neg (28)	BDG neg GM neg	29	VCZ (5) FEP (5)	–	*Klebsiella aerogenes* *Stenotrophomonas maltophilia*	Start MEM and TMP/SMX to target organisms identified. Prevented escalation of antifungals, remained on VCZ for ppx. Later confirmed *S. maltophilia* on subsequent respiratory cx.	Died
9	M/44	HFrEF	Cavitary PNA with GGO	Moderate (20)	GM neg Cx neg (17)	BDG neg GM neg	20	CRO (13) Flagyl (13)	–	*Legionella pneumophila* (184)	Start treatment for *Legionella* pneumonia.	Lost to follow up
10	M/39	OLT	Lung mass with surrounding GGO	Mild (2)	–	BDG neg GM neg	4	VCZ (2)	–	–	Continued empiric VCZ	Alive
11	M/53	DDKT	Interspersed focal consolidation and GGO with halo sign	Mild (5)	–	BDG neg GM neg	6	VCZ (5) MYC (5)	–	*R. mucilaginosa* (5156), *Prevotella melaninogenica* (3023), *Veillonella parvula* (888), *Veillonella dispar* (546), *Actinomyces odontolyticus* (485), *E. faecalis* (300)	Continued empiric VCZ	Alive
12	F/72	OHT	Nodular PNA with patchy GGO	Moderate (4)	–	BDG neg GM neg	9	IVCZ (2) TZP (2)	–	–	IVCZ discontinued day mcfDNA resulted	Alive
13	F/54	DDKT	PNA with hypoxia/fever, diffuse GGO	Mild (74)	GM neg Cx neg Pathology not sent (12)	BDG (282 pg./mL), GM neg	16	PSZ (2) CPT (2) DOX (3) MEM (5) TMP/SMX (5)	–	*R. mucilaginosa* (476), *Streptomyces cattleya* (310), *Pneumocystis jirovecii* (233)	Confirmed diagnosis, started steroids and continued TMP/SMX for *P. jirovecii.* Stopped other antimicrobials once mcfDNA resulted	Alive

a Allo HSCT, Allogeneic hematopoietic stem cell transplant; Alt, alternative; AML, acute myelogenous leukemia; AMB, liposomal amphotericin B; *A. odontolyticus*, *A. odontolyticus*; B, bloodstream infection; BAL, bronchoalveolar lavage; BDG, 1,3-β-D-Glucan, *B. ovatus*: *Bacteroides ovatus*; *C. difficile*, *Clostridioides difficile*; CLL, chronic lymphocytic leukemia; CMV, cytomegalovirus; COVID, coronavirus 2019; CPT, ceftaroline; CRO, ceftriaxone; CT, computed tomography; CX, culture; DDKT, Deceased donor kidney transplant; Dox, doxycycline; Dx, diagnosis; EBV, Epstein bar virus; *E. coli*, *Escherichia coli*; *E faecium*, *E. faecium*; *E. faecalis*, *E. faecalis*; ETT, endotracheal tube; F, female; FEP, cefepime; G, gastrointestinal infection; *G. adiacens*, *G. adiacens*; GGO, ground-glass opacities; GM, galactomannan; GVHD, graft vs. host disease; HFrEF, heart failure with reduced ejection fraction; IMI, invasive mold infection; IU, index unit; IVCZ, isavuconazole; *Klebsiella aerogenes*, *K. aerogenes*; *L. pneumophilia*, *Legionella pneumophilia*; *L. rhamnosus*, *Lacticaseibacillus rhamnosus*; LVX, levofloxacin; mcfNGS, plasma microbial cell free next generation sequencing; M, male; MEM, meropenem; MPM, molecules of microbial cell free DNA per microliter; MYC, micafungin; MV, mechanical ventilation; Neg, negative; No, number; OHT, orthotropic heart transplant; OLT, orthotropic liver transplant; ppx, prophylaxis; *P. aeruginosa*, *Pseudomonas aeruginosa*; *P. jirovecii*, *Pneumocystis jirovecii*; *P. melaninogenica*, *P. melaninogenica*; PNA, pneumonia; PO, oral; Pos, positive; PSZ, posaconazole; Pt, patient; *R. mucilaginosa*, *Rothia mucilaginosa*; *S.cattelya*, *Streptomyces cattleya*; *S. cerevisiae*, *S. cerevisiae*; *S. maltophilia*, *S. maltophilia*; *Streptococcus oralis*, *S. oralis*; SCLC, small cell lung carcinoma; SOB, shortness of breath; Sx, symptom; TMP/SMX, trimethroprim-sulfamethoxazole; TZP, piperacillin-tazobactam; VAN, vancomycin; VATS, video assisted thoracic surgery; *V. dispar*, *Veillonella dispar*; VCZ, voriconazole; *V. parvula*, *Veillonella parvula*; yr., year.

b Severity of COVID-19 infection per National Institute of Health guidelines (days from symptom onset to mcfNGS).

c Results from bronchoalveolar lavage (BAL) including all available culture results, cytology and GM results and (time from onset of symptoms to BAL) Antimicrobials used and (days of treatment prior to mcfNGS testing).

d Days from symptom onset to mcfNGS.

e Antimicrobials used at time of mcfNGS and (duration prior to testing).

f Infection known at time of testing- including organism and (source, days from diagnosis to mcfNGS testing).

g Organism detected on mcfNGS (molecules per microliter).

In the cohort, mcfNGS was positive for 1–6 organisms in 61% (8/13) patients including bacteria (n = 7), fungi (n = 2), and viruses (n = 2). Of these, 31% (4/13) tests identified organisms that were determined to be clinically significant (patients 2, 8, 9, and 13) and prompted management changes to target these organisms. This included initiation of treatment for organisms not previously covered (patients 2, 8, 9) and de-escalation of additional antimicrobials (patient 13). The two fungal organisms detected were *Pneumocystis jirovecii* and *Saccharomyces cervisiae.* The patient in which *P. jirovecii* was detected had an elevated BDG and underwent BAL, however cytopathology and *P. jirovecii* PCR were not obtained from the BAL fluid. The *P. jirovecii* detected on mcfNGS was considered clinically significant and the patient continued trimethoprim/sulfamethoxazole and prednisone that were started empirically while additional antimicrobials were de-escalated. *S. cervisiae* was detected in a patient with acute alcoholic hepatitis and was thought to represent colonization rather than a true fungal pneumonia; the absence of any pathogenic molds assisted with decision making regarding listing for liver transplant. This patient had a positive BDG which is of unclear significance, but this can occur in the setting of infections due to *S. cervisiae.* The patient was continued on micafungin for antifungal prophylaxis perioperatively. Overall, our cohort had an 85% (11/13) concordance rate, including negative and positive results, for fungal pathogens with the available microbiologic data, including serum fungal biomarkers and BAL studies. Of the two patients with discordant results, one had a positive serum GM without fungal organisms detected by mcfNGS, and one was treated empirically for IMI. Both patients had a BAL with negative GM and cultures. The patient with a positive serum GM had a negative BAL GM, however had hyphae on cytology and therefore antifungals were changed to treat a possible IMI. However, the patient later had a video assisted thoracic surgery (VATS) guided lung biopsy that showed organizing pneumonia but no fungal elements or cultures positive that would be consistent with IMI. Therefore, the serum GM was considered a false positive result. For the other patient, empiric antifungals were de-escalated after the mcfNGS returned without fungal organisms detected. There were no patients who had fungal organisms detected on culture in this cohort.

Three patients had bacterial organisms detected on mcfNGS that were determined to be clinically significant. These included: *Rothia mucilaginosa* (patient 2), *Stenotrophomonas maltophilia* and *Klebsiella aerogenes* (patient 8), and *Legionella pneumophilia* (patient 9). Patient 8 did not have any positive cultures at the time of testing but *S. maltophilia* was recovered in subsequent respiratory cultures. The other organisms were not identified on orthogonal testing. None of the patients were on adequate antibiotic therapy for these organisms. Patient 2 was started on cefdinir for *R. mucliaginosa* with partial improvement in hypoxia. However, they were later diagnosed with small cell lung carcinoma via biopsy and therefore the significance of this remains unclear. Patient 8 was started on meropenem and trimethoprim sulfamethoxazole for *K. aerogenes* and *S. maltophilia.* Patient 9 was treated with azithromycin for the *Legionella pneumophilia* detected on mcfNGS. They had a pulmonary mass with negative BAL cultures and negative urine legionella antigen. The treating physicians recommended biopsy was given that it was unclear if *Legionella* was the causative organism, but the patient declined this and was subsequently lost to follow up. Three patients (23%) had results that were determined not to be clinically significant (patients 4, 7, and 11), and one (8%) had mixed results with previously identified pathogens and commensal organisms (patient 6).

Overall, mcfNGS results led to a change in clinical management in 85% (11/13) of patients. In one case, the detection of *Pneumocystis jirovecii* confirmed the clinical diagnosis and led to the de-escalation of additional empiric antimicrobials. The lack of fungal organisms detected by mcfNGS prompted the de-escalation or discontinuation of empiric antifungals in 31% (4/13) of cases and prevented the contingent escalation of therapy in 23.1% (3/13). None of these patients later developed confirmed IMI after antifungal de-escalation. These data also shortened the duration of therapy in 8% (1/13) of cases. Additionally, initiation of antimicrobial therapy occurred in 23% (3/13) to target organisms identified. One test also assisted in the plan to proceed with organ transplantation. Despite the absence of fungi on mcfNGS, empiric antifungals were continued as prophylactic therapy for 23% (3/14) due to their ongoing high risk of IMI.

## Discussion

In a cohort of largely immunocompromised patients admitted with COVID-19 who were undergoing evaluation for secondary pneumonia, mcfNGS identified a causative pathogen in 4 of 13 patients and potentially influenced clinical management in 11/13. In this study, it identified respiratory microorganisms including *Pneumocystis jirovecii*, *Legionella pneumophilia, R. mucilaginosa*, *Stenotrophomonas maltophila* and *K. aerogenes* that were determined to be clinically relevant based on the assessment of the patient, even though orthogonal testing was unable to corroborate the findings.

In addition, mcfNGS identified various organisms that were determined not to be clinically significant, including *Saccharomyces cervisiae.* In these cases, mcfNGS was still helpful in assisting with antimicrobial de-escalation or prevented contingent antifungal escalation. Prior studies demonstrate a negative predictive value of 81–99% for fungal infections by mcfNGS, although recent studies indicate that this may be as low as 31% for *Aspergillus* spp. specifically [16]. In our cohort, the lack of detection of fungal organisms by mcfNGS in conjunction with fungal biomarker profiles and clinical findings all collectively conferred assurance for providers to address other potential etiologies of each patient’s secondary worsening and assisted with changes in antifungal management in 6 cases.

In the existing literature, the concordance of mcfNGS with microbiologic diagnostic methods range from 22–100% depending on preceding antimicrobial use, timing, site of infection and population, with a higher concordance in immunocompromised populations [22]. The specificity and clinical impact that is reported in the literature is also variable. In one multicenter retrospective review of mcfNGS used for all indications, even though 61% of Karius tests were positive, only 7.3% of results led to a positive impact and 3.7% led to a negative clinical impact, including unnecessary diagnostic interventions and treatment. [23] However, 33% of patients had a pre-established diagnosis through conventional testing before mcfNGS was performed [23]. In contrast, other studies have shown a positive clinical impact in 47% [24]. These differences are likely due to differences in patient selection, including pre-established diagnosis and timing of testing, and involvement in Infectious Disease specialists to guide test ordering and interpretation. The turnaround time of the test, which is 26 h from receipt of the sample, may also influence clinical impact.

There are only three studies published in the literature to date evaluating the use of plasma mcfNGS to evaluate secondary pneumonia after COVID-19. Hoenigl et al. [19] evaluated plasma samples in patients with COVID-19 associated respiratory failure admitted to the intensive care unit and found that mcfNGS had a sensitivity of 83% and specificity of 97% in patients with probable CAPA, including a case where all other blood biomarkers and tests for aspergillosis were negative. Kitsios et al. [18] evaluated 15 critically ill patients with COVID-19 with mcfNGS and found high mortality associated with total mcfDNA molecules per microlite. In the 11/15 of patients with suspected secondary pneumonia, those with positive respiratory cultures also had high MPM values of bacterial pathogens and 2/8 of the remaining patients with suspected secondary infections had probable pathogens detected. There were 4 patients who had pathogens detected that were not on antibiotics, 2 of which died from shock and multiorgan failure. It is possible that they had an undiagnosed secondary infection contributing to these outcomes given the organisms detected, but it is difficult to distinguish this from translocating colonizing flora. Finally, Lee et al. [25] found a decreased positive agreement of mcfNGS in the diagnosis of pulmonary aspergillosis in patients with COVID compared to those with hematologic malignancies (92.3% vs. 50%), however this was using a different laboratory-developed test in Korea, and therefore the results may not be comparable due to differences in testing techniques.

This study is limited as a case series and due to potential selection bias for patients in which mcfNGS was performed, which was done at the recommendation of an Infectious Diseases physician. It also does not provide an understanding of the population that did not receive mcfNGS testing. In addition, most of the patients selected did not have diagnostic procedures such as bronchoscopy performed, which may have been due to clinical status. As a retrospective study, the ability to interpret the impact of mcfNGS results on patient care was limited to the clinical data available via chart review. The determination of pathogenicity was subjective, based on individual assessment of the available clinical data which was often not comprehensive. While this represents a realistic scenario of patients unable to undergo invasive procedures to obtain a diagnosis, the true significance of the results remains unclear. In addition, the clinical utility of the test was also a subjective assessment, which as a retrospective study was limited to the documentation available and timing of antimicrobial changes. Larger, prospective, multi-center studies should further assess how mcfNGS results, including those that are negative, are used in clinical decision-making prospectively.

In conclusion, in our series, mcfNGS was used to evaluate secondary pneumonia in individuals with COVID-19. The results facilitated the diagnosis of secondary opportunistic infections including *Pneumocystis* and *Legionella* and the de-escalation of empiric antimicrobials. This testing may have a role in the secondary pneumonia in patients with COVID-19. However, given the low sensitivity of mcfNGS reported for *Aspergillus* spp., providers must be cautious in interpreting results in conjunction with additional clinical findings.

## Data Availability

The data that support the findings of this study are not openly available due to reasons of sensitivity and are available from the corresponding author upon reasonable request.
